# The Status of SOX2 Expression in Gastric Cancers with Induction of CDX2 Defines Groups with Different Genomic Landscapes

**DOI:** 10.3390/genes16030279

**Published:** 2025-02-26

**Authors:** Ioannis A. Voutsadakis

**Affiliations:** 1 Algoma District Cancer Program, Sault Area Hospital, 750 Great Northern Road, Sault Ste. Marie, ON P6B 0A8, Canada; ivoutsadakis@yahoo.com or ivoutsadakis@nosm.ca; 2 Section of Internal Medicine, Division of Clinical Sciences, Northern Ontario School of Medicine, Sudbury, ON P3E 2C6, Canada

**Keywords:** gastric adenocarcinoma, intestinal metaplasia, intestinal type, transcription factors, SOX2

## Abstract

Background: Gastric adenocarcinoma is a highly lethal neoplasm with a short survival especially when metastatic. Few effective treatments are available for the control of the disease and palliation of patients with metastatic gastric cancer. Although progress has been made in the elucidation of molecular pathways invoked in gastric carcinogenesis, this knowledge has not yet led to major breakthroughs, in contrast to several other types of cancer. The role of stem cell transcription factors SOX2 and CDX2 is of particular interest in the pathogenesis of gastric cancer. Methods: The cohort of gastric adenocarcinomas from The Cancer Genome Atlas (TCGA) was interrogated and two groups of gastric cancers, with CDX2 induction and SOX2 suppression on the one hand and with CDX2 induction and SOX2 maintained expression on the other hand were retained. The induction of expression of the two transcription factors was defined as a mRNA expression z score compared with normal samples above zero. The two groups were compared for clinical-pathologic and genomic differences. Results: Among gastric cancers with up-regulated CDX2 mRNA, cancers with suppressed SOX2 mRNA were slightly more numerous (55.9%) than those with a maintained SOX2 expression. The SOX2 suppressed group had a higher prevalence of MSI high cancers (30.9% versus 10%) and of cases with high tumor mutation burden (35% versus 12.4%) than cancers with a SOX2 maintained expression, which presented more frequently high Chromosomal Instability (CIN). The group with SOX2 suppression had higher rates of mutations in many gastric cancer-associated genes such as epigenetic modifiers *ARID1A*, *KMT2D*, *KMT2C*, and *KMT2B*, as well as higher rates of mutations in genes encoding for receptor tyrosine kinases *ERBB4* and *FGFR1*. On the other hand, *TP53* mutations and amplifications in *MYC*, *ERBB2*, and *CCNE1* were more common in the group with a maintained expression of SOX2, approaching significance for *MYC*. Conclusions: Notable differences are present in the genomic landscape of CDX2-induced gastric cancer depending on the level of expression of SOX2 mRNA. Despite this, SOX2 mRNA expression levels were not prognostic.

## 1. Introduction

Gastric adenocarcinomas are prevalent cancers of the gastrointestinal tract and are among the top causes of cancer mortality across populations [1]. Globally over 950,000 new cases and 650,000 deaths related to gastric cancer are recorded annually, making the disease the fifth most prevalent and fifth most lethal cancer [1]. Despite advances in treatment, metastatic disease is almost invariably lethal. Most patients with metastatic gastric adenocarcinomas are treated with palliative chemotherapy, but the median survival is grim. In the recent SPOTLIGHT and GLOW trials that tested the addition of the anti-claudin 18.2 monoclonal antibody zolbetuximab to combination chemotherapy in the first-line therapy of patients with metastatic gastric cancer, the median overall survival in the control arms of both trials was about 12 months [2,3]. The addition of targeted therapies, such as zolbetuximab or trastuzumab in patients with gastric cancer who express the respective targets, slightly increase survival outcomes [2,3,4]. Another target recently added in the armamentarium of gastric carcinoma therapeutics is the FGFR2a receptor, which can be effectively inhibited with the monoclonal antibody bemarituzumab [5]. Bemarituzumab with combination FOLFOX chemotherapy improved survival outcomes in patients with gastric cancer with a high expression of the receptor to a median of 19.2 months, from a median of 13.5 months with FOLFOX alone. Immunotherapy with immune checkpoint inhibitors is also effective in prolonging survival in a sub-set of patients with mismatch repair deficiency or expression of the ligand PD-L1 [6]. The long-term survival of most patients is not significantly improved even with these treatments due to development of resistance. Development of new treatments is a pressing necessity and awaits a better understanding of the molecular pathogenesis of gastric cancer. Studies seeking to elucidate the molecular landscape of cancers led by The Cancer Genome Atlas (TCGA) consortium and other investigators have provided a dearth of genomic data that will help in this task [7].

Two main histologic types of gastric adenocarcinomas have been distinguished, the intestinal type and the diffuse type [8]. The two types have different morphology, metastatic predilection, and underlying genomic lesions [9,10]. Various prevalent injurious factors in the upper gastrointestinal tract, such as bile acid and acidity exposure and *helicobacter pylori* (*h. pylori*) infection, promote gastric epithelial metaplasia to intestinal epithelium, which becomes the background for increasing levels of dysplasia [11]. Intestinal-type gastric carcinomas arise from these dysplastic lesions in a cascade first proposed by Correa [12,13]. A similar cascade is observed in the lower esophagus, where Barrett’s esophageal lesions are the phenotypic intermediates, although differences exist most probably related to the different most prevalent insults in the two locations [14]. The transcription factor CDX2 (Caudal related Homeobox transcription factor 2) is an important determinant of the intestinal phenotype and is up-regulated in gastric epithelia that have undergone metaplasia. It is also expressed in sub-sets of gastric cancers [15]. In contrast, SOX2 (SRY-related HMG Box 2), a stem cell factor that specifies the normal foregut and is expressed in normal gastric epithelium, is down-regulated in sub-sets of gastric cancers.

The current investigation examines gastric adenocarcinomas that have acquired CDX2 mRNA up-regulation for their concomitant expression of SOX2 and compares CDX2 up-regulated cancers that maintained SOX2 expression with CDX2 up-regulated cancers that have acquired concomitant SOX2 suppression. The two groups are compared for defining clinical and pathologic characteristics and other relevant genomic features in their respective landscapes, aiming at describing therapeutically targetable attributes.

## 2. Methods

The gastric cohort of The Cancer Genome Atlas (TCGA) consisting of 440 patients with their genomic samples was the basis of the study [7]. A sub-cohort consisting of 220 patients with an up-regulation of CDX2 mRNA, defined as an mRNA expression z score compared with normal samples of above 0, were included in the current study. Two groups were constructed based on SOX2 mRNA expression (SOX2 mRNA expression z score compared with normal samples above or below 0). The level of mRNA expression was calculated and normalized from RNA Seq data with the RSEM (RNA-Seq by Expectation Maximization) algorithm which can be employed without the need of a reference genome [16]. TCGA has analyzed genomic data using a whole exome next generation sequencing platform and contains data on single nucleotide variations, copy number alterations, and structural variants [7]. Single nucleotide variants were called according to various algorithms used in participating institutions, and copy number alterations were estimated with the GISTIC (Genomic Identification of Significant Targets In Cancer) algorithm [17,18].

Clinical, pathologic, and genomic parameters of the TCGA gastric cancer cohort were analyzed using the cBioportal for cancer genomics platform (www.cbioportal.org) [19,20]. cBioportal is an open access platform and contains a wide range of clinical, pathologic, and genomic data from studies performed by investigators worldwide and made publicly available. The landmark studies from TCGA and from other groups can be interrogated for genomic alterations of interest with a minimal requirement for bioinformatics expertise [19,20]. For the current investigation, two groups of gastric cancers, with CDX2 mRNA-induction/SOX2-maintained expression and with CDX2 mRNA-induction/SOX2-suppressed expression were constructed and evaluated.

The OncoKB knowledge base (www.oncokb.org accessed 16 January 2025) was used for the annotation of mutations of interest [21,22]. OncoKB provides a manually curated assignment of pathogenicity of specific cancer-associated mutations and classifies mutations to pathogenic/likely pathogenic, likely neutral, and of unknown significance.

Statistical analyses were performed with the Student’s *t* test or Analysis of Variance (ANOVA) for continuous parameters and with the Fisher’s exact test or the χ^2^ test for categorical data. Survival was visualized with the construction of Kaplan–Meier curves, and survival comparisons were performed with the Log Rank test. Statistical significance was set at the level of *p* < 0.05. Corrections for multiple comparisons were made with the Benjamini–Hochberg procedure.

## 3. Results

The majority of patients in the gastric adenocarcinoma cohort study of TCGA (240 of 440 patients or 54.5%) had an up-regulation of CDX2 (mRNA expression z score compared with normal samples above zero). Of the 240 patients with CDX2 induction, 220 patient samples had data on the mRNA expression of SOX2. Ninety-seven of those (44.1%) retained expression of SOX2 mRNA, as defined by a z score compared with normal samples above zero, and 123 patients (55.9%) displayed suppression of SOX2 mRNA expression, as defined by a z score compared with normal samples below zero. The cut-off of these z scores for both CDX2 and SOX2 mRNA expressions was selected arbitrarily at zero, as the distribution of these expressions was continuous with no clear expression level defining a gap (Supplemental Figure S1). Patients in the group with retained SOX2 expression were younger (mean age of 64.2 years old) than patients with suppressed SOX2 expression (mean age of 67.7 years old and a Student’s *t* test with *p* = 0.01, in Table 1). In addition, early stage disease (stage I and II) was more common (56.2% of cases) in the group with SOX2 suppression compared with 39.5% in the group with maintained SOX2 expression (χ^2^ test with *p* = 0.04). There were no differences between the two groups in the prevalence of early onset disease (at an age younger than 50 years old), gender distribution, and histologic subtype and grade (Table 1). The group of gastric cancers with retained SOX2 expression belonged more often (74.5%) to the chromosome instability (CIN)-high genomic category compared with SOX2-suppressed group where CIN cancers constituted 54.6% of the group (Table 2). In the latter group, microsatellite instability (MSI) was more common (30.9% of cases) compared with 10% of cases in the group with retained SOX2 expression. Consistent with the genomic categories, cases with a high tumor mutation burden (TMB) above 10 mutations/Mb had a higher prevalence in the group with suppressed SOX2 mRNA, while CIN metrics AS and FGA were more often high in the group with retained SOX2 expression (Table 2).

**Figure 1:**
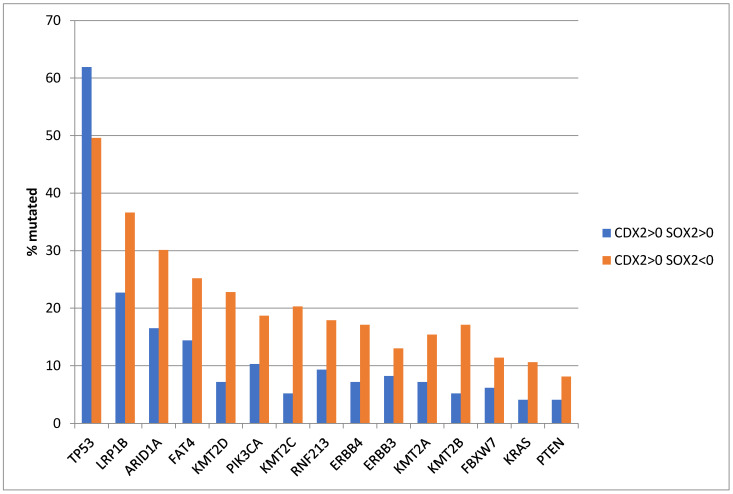
Prevalence of the most frequent gastric adenocarcinoma mutations in patients with CDX2 induction (mRNA expression z score relative to normal samples above 0) and maintained SOX2 expression (mRNA expression z score relative to normal samples above 0, *n* = 97) or suppressed SOX2 expression (mRNA expression z score relative to normal samples below 0, *n* = 123). Data are from The Cancer Genome Atlas (TCGA).

The most frequently mutated tumor suppressor gene in gastric and many other cancers, *TP53*, encoded for p53, was commonly mutated in both groups with a higher prevalence in the group with SOX2 maintenance of expression (61.9% versus 49.6% in the group with suppressed SOX2 expression) which approached statistical significance (Fisher’s exact test with *p* = 0.07). In contrast, almost all other common mutations of gastric cancer were more prevalent in the group with SOX2 suppression (Figure 1). Among frequently mutated genes, several encoding for epigenetic modifiers displayed significant higher rates in the SOX2-suppressed group (Figure 1 and Table 3). Several prominent members of the SWI/SNF chromatin remodeling complex were in this category, with *ARID1A* being the gene with the highest prevalence of mutations. *ARID1A* was mutated in 30.1% of cases in the SOX2-suppressed group and in 16.5% of cases in the group with maintained SOX2 expression (Fisher’s exact test with *p* = 0.02 in Figure 1 and Table 3). In addition, mutations in histone methyltransferases *KMT2B*, *KMT2C*, and *KMT2D* were more frequent (17.1%, 20.3%, and 22.8% of cases, respectively) in the group with SOX2-suppressed expression than in the group with retained SOX2 expression (5.2%, 5.2%, and 7.2% of cases, respectively with Fisher’s exact tests with *p* = 0.006, 0.001, and 0.001, respectively, in Figure 1 and Table 3). The comparisons for *KMT2C* and *KMT2D* maintained statistical significance after correction for multiple comparisons (Table 3). Genes encoding for receptor tyrosine kinase pathways were also more prevalent in the SOX2-suppressed group with the difference reaching significance for mutations in *ERBB4* (17.1% of the cases in the SOX2-suppressed group and 7.2% of the cases in the group with maintained SOX2 expression with Fisher’s exact test with *p* = 0.04) and approaching significance for *PIK3CA* (18.7% of the cases in the SOX2-suppressed group and 10.3% of the cases in the group with maintained SOX2 expression with Fisher’s exact test with *p* = 0.09) (Figure 1 and Table 4). Mutations in frequently mutated genes of gastric cancer, included in Figure 1, occurring in the MSI-high sub-set of CDX2-induced, SOX2-suppressed gastric cancers, were in the majority (53.2%) deemed pathogenic in the evaluation performed by the OncoKB knowledgebase. Other less frequent mutations in receptor tyrosine kinases were also more prevalent in the group with suppressed SOX2 mRNA expression (Table 4). Mutations of several genes encoding for proteins involved in DNA damage response and repair were also more prevalent in gastric cancers with suppressed SOX2 mRNA expression, although the frequency of mutations in each individual gene was low (Table 5). None of the differences in the prevalence of mutations in genes encoding for receptor tyrosine kinase pathway members or involved in DNA damage response and repair were statistically significant after correction for multiple comparisons. MMR-related gene mutations showed no significant differences between the two groups, suggesting that the high rate of MSI in the SOX2-suppressed group is due to epigenetic alterations or post-translational pathway dysfunction (Table 6).

**Figure 2:**
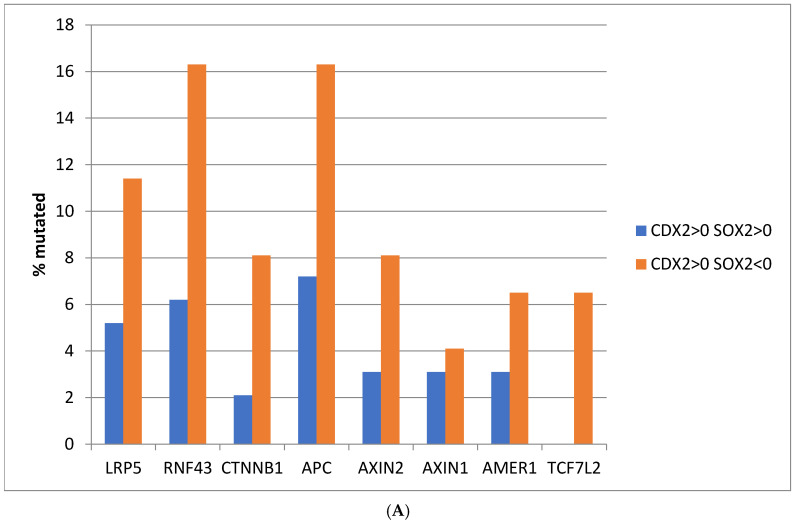

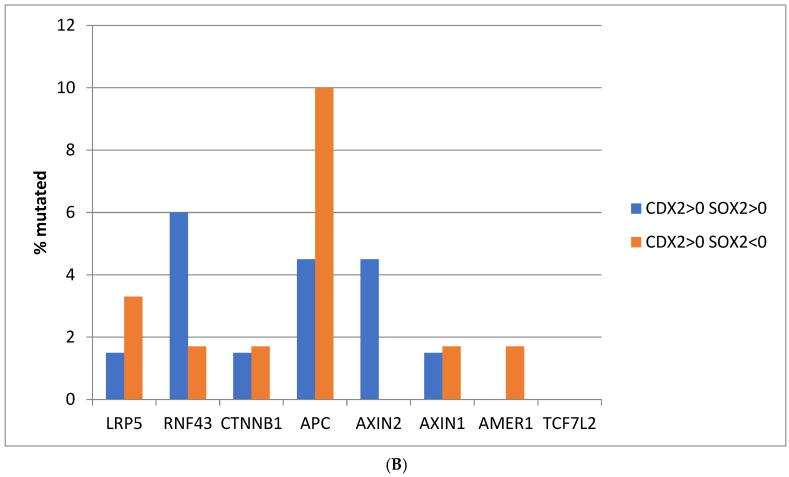
(**A**) Prevalence of mutations of genes of the WNT/β-catenin/APC pathway in patients with CDX2 induction (mRNA expression z score relative to normal samples above 0) and maintained expression (mRNA expression z score relative to normal samples above 0, *n* = 97) or suppressed expression (mRNA expression z score relative to normal samples below 0, *n* = 123) of SOX2. (**B**) Comparison restricted in the subgroups with Chromosomal Instability (CIN). Data are from The Cancer Genome Atlas (TCGA).

The WNT/APC/β-catenin pathway is a prominent cancer-related signaling pathway deregulated in gastrointestinal cancers and a regulator of CDX2 expression. Mutations in components of the pathway were more prevalent in gastric cancers with suppressed SOX2 expression, compared to the group with maintained expression of SOX2, with the difference being significant for ligase *RNF43*, which showed mutations in 16.3% of cancers with suppressed expression of SOX2 and in 6.2% of cancers with maintained SOX2 expression (Fisher’s exact test with *p* = 0.03) and, for *TCF7L2*, which was mutated in 6.5% of cases with suppressed expression of SOX2, and in none of the cases with maintained expression of SOX2 (Fisher’s exact test with *p* = 0.009, in Figure 2A). Mutations in APC were also frequent in gastric cancers with CDX2 induction and SOX2 suppression (16.2% of cases), and the difference in prevalence compared with SOX2-maintained-expression cancers approached significance (Fisher’s exact test with *p* = 0.06). The differences observed in the mutation rates of WNT/APC/β-catenin pathway components in the two groups differing in SOX2 expression was in a significant degree due to the high mutation rate in MSI-high cancers, which were more prevalent in cancers with suppressed SOX2 mRNA expression. A comparison restricted in CIN cancers from the two groups disclosed only minor differences that did not reach statistical significance (Figure 2B). The proportion of pathogenic mutations in the examined WNT/APC/β-catenin pathway genes of the MSI-high subgroup of CDX2-induced, SOX2-suppressed gastric cancers was 28.6% (22 of 77 mutations), while the rest were of unknown significance, according to the evaluation in the OncoKB knowledgebase.

**Figure 3:**
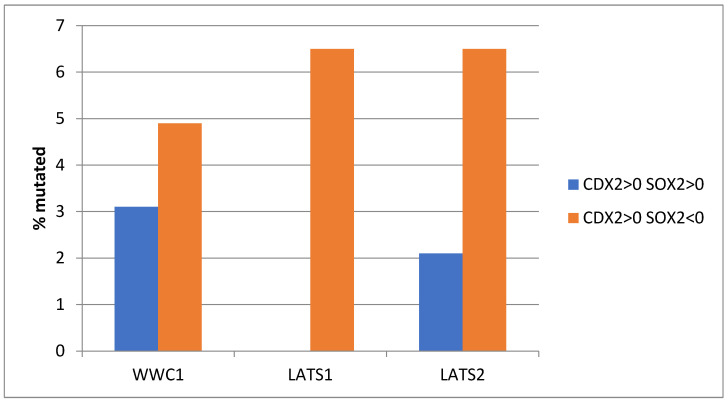
Prevalence of mutations of genes of the Hippo pathway in patients with CDX2 induction (mRNA expression z score relative to normal samples above 0) and maintained expression (mRNA expression z score relative to normal samples above 0, *n* = 97) or suppressed expression (mRNA expression z score relative to normal samples below 0, *n* = 123) of SOX2. Data are from The Cancer Genome Atlas (TCGA).

Another pathway with a significant role in CDX2 regulation, the Hippo pathway, showed a lower prevalence of mutations in gastric cancers. One of the core regulators of the pathway, kinase LATS1, is mutated in 6.5% of cases with suppressed SOX2 mRNA expression and in no cases with maintained SOX2 expression (Fisher’s exact test with *p* = 0.009, in Figure 3). Two other key regulators of the Hippo pathway, LATS2 and WWC1 (also called KIBRA), showed mutations in 6.5% and 4.9% of the cancers in the group with SOX2 suppression, which was higher than in the group with maintained SOX2 expression (2.1% and 3.1%, respectively) but without attaining statistical significance (Fisher’s exact test with *p* = 0.19 and 0.73, respectively, in Figure 3).

**Figure 4:**
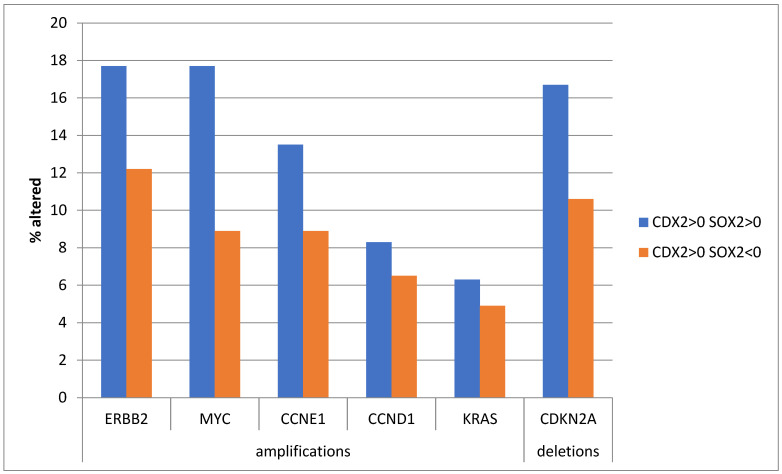
Prevalence of frequent gastric adenocarcinoma copy number alterations in patients with CDX2 induction (mRNA expression z score relative to normal samples above 0) and maintained expression (mRNA expression z score relative to normal samples above 0, *n* = 97) or suppressed expression (mRNA expression z score relative to normal samples below 0, *n* = 123) of SOX2. Data are from The Cancer Genome Atlas (TCGA).

In contrast to prominent mutations, copy number alterations showed a trend for higher prevalence in the group with SOX2-maintained expression (Figure 4). The difference approached statistical significance for *MYC* amplifications, which were present in 17.7% of cases with SOX2-maintained expression and 8.9% of cases with suppressed SOX2 mRNA expression (Fisher’s exact test with *p* = 0.06, in Figure 4). A similar high prevalence of *ERBB2* amplifications and *CDKN2A* deletions was observed in the group with maintained SOX2 mRNA expression.

**Figure 5:**
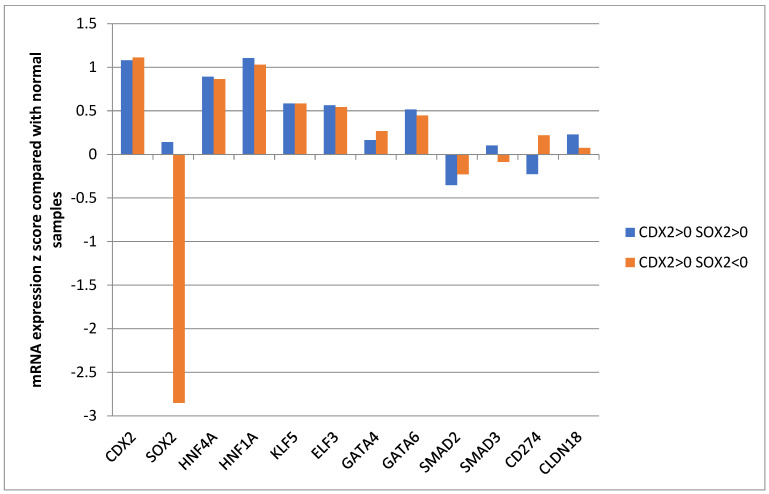
Expression of transcription factors involved in the CDX2/SOX2 network in patients with CDX2 induction (mRNA expression z score relative to normal samples above 0) and maintained expression (mRNA expression z score relative to normal samples above 0) or suppressed expression (mRNA expression z score relative to normal samples below 0) of SOX2. Data are from The Cancer Genome Atlas (TCGA).

CDX2 is part of a network of transcription factors, including SOX2, HNF4A, HNF1A, KLF5, ELF3, GATA4, GATA6, and the effectors of the TGFβ pathway, SMAD2 and SMAD3, whose coordinated expression and function is important for tissue specification. Among these factors, neither the expression of CDX2 nor of any other factor differed significantly between the groups with SOX2 suppression and maintained SOX2 expression (Figure 5).

**Figure 6:**
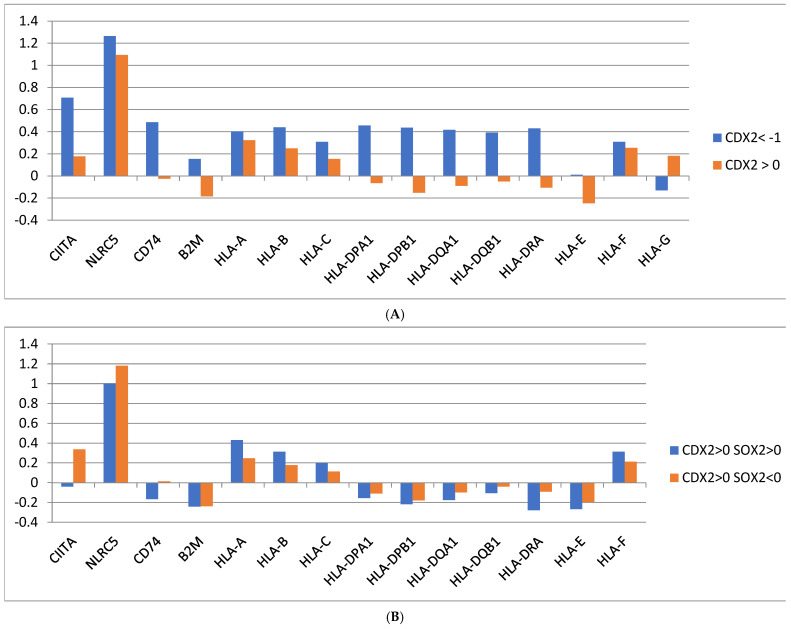
(**A**) Expression of Human Leukocyte Antigen (HLA) class II proteins in patients with CDX2 induction (mRNA expression z score relative to normal samples above 0) compared with suppressed CDX2 (mRNA expression z score relative to normal samples below −1). (**B**) Expression of Human Leukocyte Antigen (HLA) class II proteins in patients with CDX2 induction (mRNA expression z score relative to normal samples above 0) and maintained expression (mRNA expression z score relative to normal samples above 0) or suppressed expression (mRNA expression z score relative to normal samples below 0) of SOX2. Data are from The Cancer Genome Atlas (TCGA).

Gastric cancers with induced CDX2 mRNA expression (independent of SOX2 expression) showed significantly lower expression of Human Leukocyte Antigen (HLA) class II proteins HLA-DPA1, HLA-DPB1, HLA-DQA1, HLA-DQB1, and HLA-DRA (Student’s *t* test with *p* < 0.0001 for all comparisons) compared with gastric cancers with maintained CDX2 suppression, while HLA class I proteins HLA-A, HLA-B, and HLA-C displaced smaller differences between the groups (Figure 6A). In addition, the master regulator of HLA class II proteins, CTAII, and the invariant chain protein associated with HLA class II proteins, CD74, displayed a lower expression in CDX2-induced gastric cancers compared with cancers that maintained CDX2 mRNA suppression (Student’s *t* test with *p* < 0.0001 for both comparisons). However, the two subgroups of CDX2-induced gastric cancers, with SOX2-maintained mRNA expression and SOX2 mRNA suppression showed minimal differences in member proteins of the immune presentation machinery (Figure 6B).

**Figure 7:**
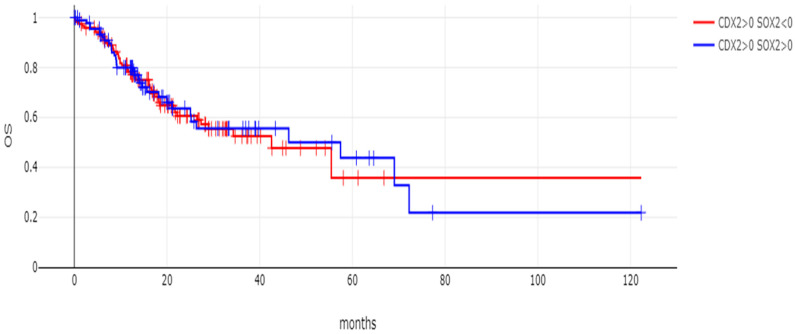
OS of patients with CDX2 induction (mRNA expression z score relative to normal samples above 0) and maintained expression (mRNA expression z score relative to normal samples above 0, *n* = 96) or suppressed expression (mRNA expression z score relative to normal samples below 0, *n* = 121) of SOX2. Log Rank *p* = 0.81.

**Figure 8:**
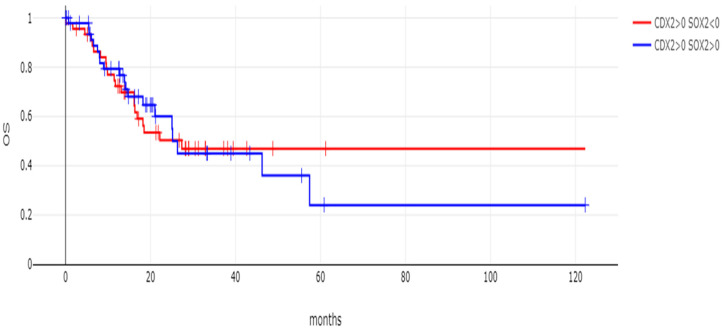
OS of stage 3 patients with CDX2 induction (mRNA expression z score relative to normal samples above 0) and maintained expression (mRNA expression z score relative to normal samples above 0, *n* = 51) or suppressed expression (mRNA expression z score relative to normal samples below 0, *n* = 45) of SOX2. Log Rank *p* = 0.94.

**Figure 9:**
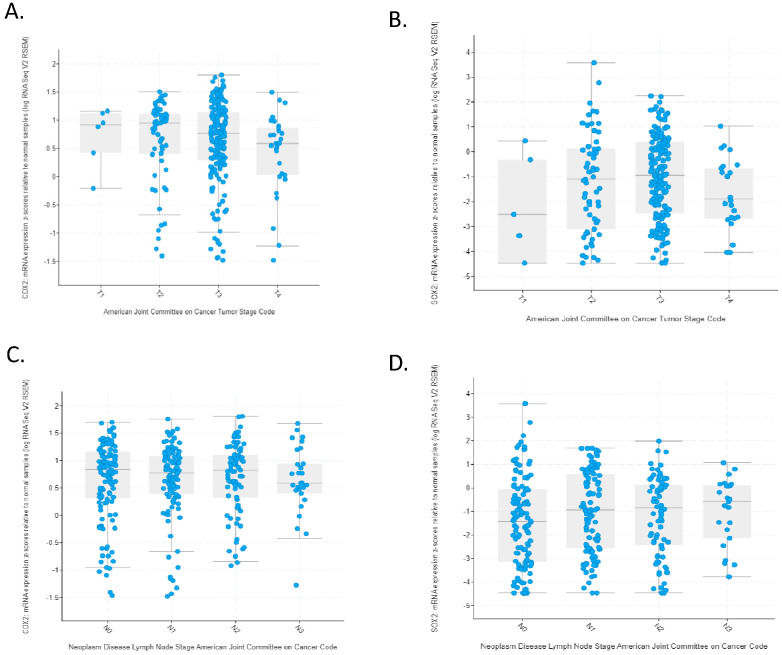
mRNA expression of CDX2 and SOX2 according to tumor T and N stage. (**A**) mRNA expression of CDX2 according to tumor T stage. (**B**) mRNA expression of SOX2 according to tumor T stage. (**C**) mRNA expression of CDX2 according to tumor N stage. (**D**) mRNA expression of SOX2 according to tumor N stage.

The overall survival of the two groups of gastric cancers with CDX2 induction and SOX2-maintained expression or SOX2 mRNA suppression did not differ significantly, with the survival curves of the two groups virtually overlapping, suggesting that, despite being associated with differences in genomic lesions, SOX2 mRNA levels did not convey prognostic information (Log Rank *p* = 0.81, Figure 7). Similarly, no difference in the overall survival of the two groups was observed when the comparison was restricted to stage 3 cancers (Log Rank *p* = 0.94, Figure 8). In addition, in contrast to CDX2 mRNA levels which were not associated with the T stage of tumors (one-way ANOVA with *p* = 0.98, shown in Figure 9A), SOX2 mRNA levels were associated with the tumor T stage (one-way ANOVA with *p* = 0.03, shown in Figure 9B). However, there was no linear tendency in these levels with T stages, as stage T1 and T4 had a higher mean SOX2 mRNA expression than T2 and T3 tumors (Figure 9B). Neither CDX2 mRNA expression levels nor SOX2 mRNA levels were associated with the N stage of tumors (one-way ANOVA with *p* = 0.63 and *p* = 0.28, respectively, Figure 9C,D).

## 4. Discussion

Gastric cancers frequently arise in a background of intestinal metaplasia, which represents the response of the gastric mucosa to various noxious insults from the environment, such as acidity, biliary acids, and *h. pylori* infection, through a stepwise progression of states as described in the Correa cascade [23]. Notably, carcinogenic stimuli may co-operate in neoplasia induction. For example, deoxycholic acid, in conditions of iron deficiency, promotes the ability of *h. pylori* to act as a carcinogen through facilitation of the entrance of oncogenic protein CagA in epithelial cells [24]. Intestinal metaplasia is, however, significantly more common than carcinomas, suggesting that only a small sub-set of metaplastic mucosa gives rise to malignancy. The pathogenic cascade is similar in the lower esophagus, where Barrett’s alterations are significantly more frequent than associated adenocarcinomas both in the presence and in the absence of gastroesophageal reflux symptoms [25]. Notably, and importantly for the pathophysiologic implications of the neoplastic cascade, incomplete (colon-type) intestinal metaplasia presents a higher risk for progression to cancer than complete (small-intestine-type) intestinal metaplasia suggesting that the probability of cancer development varies in different histologic morphologies [26]. In a micro-array study of the metaplasia/dysplasia/cancer cascade, markers of both intestinal and gastric epithelia, were commonly co-existent in precancerous lesions, and intestinal markers showed a less robust expression in carcinomas [27].

CDX2 has an important role in the specification of the lower gastrointestinal tract and, although it is not normally expressed in the stomach, it is induced in intestinal metaplasia and remains expressed in a sub-set of gastric carcinomas [28]. On the other hand, SOX2, which is a specification transcription factor for the foregut and is normally expressed in gastric epithelia, becomes often suppressed in gastric carcinomas [29]. The change of SOX2 expression is also observed in the mucosa surrounding the tumor, suggesting that, similarly to CDX2 changes, SOX2 changes precede the development of cancer. The interplay of expressions of the two factors is evocative of a reverse regulation [30]. Indeed, a reverse regulation of SOX2 and CDX2 was observed in gastric cells exposed to *h. pylori* infection and Bone Morphogenic Protein (BMP) pathway signaling, which both promote CDX2 expression and SOX2 down-regulation [31]. A similar interplay and reciprocal expression of CDX2 and SOX2 is observed in the lower esophagus during progression to the metaplastic epithelium of Barrett’s esophagus and adenocarcinoma, despite the lack of involvement of *h. pylori* in this location, suggesting that other triggers are able to promote the metaplasia to dysplasia to the cancer cascade in this case [32]. Bile acid exposure of gastric cells can suppress SOX2 expression and up-regulate CDX2 expression [33]. The mechanism of SOX2 suppression was the induction of miR-21, a de-stabilizer of SOX2 mRNA. Forced over-expression of SOX2, in addition to the suppression of CDX2, suppresses other factors associated with intestinal metaplasia, such as KLF4 and HNF4α [33]. Despite these reverse regulations of CDX2 and SOX2, a study of gastric carcinoma has not found a reverse association of the two factors when examined by IHC staining, suggesting retained expression of both factors due to deregulation of the networks that physiologically determine tissue identities [34].

In the research presented here, the influence of the level of mRNA expression of transcription factor SOX2 in the sub-set of gastric adenocarcinomas with over-expression of CDX2 was examined. Key differences discovered were an older age and earlier stage in patients with suppressed SOX2, who also had a higher percentage of MSI-high cancers and lower prevalence of CIN cancers. These observations imply that CDX2 induction during intestinal metaplasia and carcinogenesis in the stomach takes place independently of SOX2 suppression, and CDX2 may be effectively induced in some cancers which maintain some level of the physiologic expression of the upper gastrointestinal tract specification factor, SOX2. However, SOX2 suppression may be permissive for a sub-set of gastric cancers with CDX2 induction to acquire mutations in epigenetic modifier genes, including *ARID1A*, *KMT2D*, *KMT2C*, *KMT2A*, and *KMT2B*. Epigenetic gene mutations may promote acquisition of defects associated with microsatellite instability, such as promoter methylation of *MLH1*. This leads to a high TMB development in these cancers, which also acquire increased levels of mutations in several other genes associated with gastric cancer, such as in receptor tyrosine kinase pathways and the WNT/APC/β-catenin pathway. In contrast, SOX2-expression maintenance commits most CDX2-induced cancers to the CIN pathway, which is characterized by more frequent mutations in the tumor suppressor *TP53* gene and increased frequency of copy number alterations.

The increased rate of mutations in the WNT/APC/β-catenin pathway observed in CDX2-induced gastric cancers with SOX2 suppression may serve to reinforce CDX2 expression in these cancers, as WNT signaling is an inducer of CDX2 transcription [35]. Other signals are also critical for the induction of CDX2 in cells of the lower gastrointestinal tract where the factor is normally expressed and include the hippo pathway, the nuclear receptor transcription factor FXR (Farnesoid X Receptor), and modifications of the microenvironment [36,37]. These additional signals may also play a role in gastric carcinomas, given that WNT/APC/β-catenin pathway-activated mutations are not universally observed in CDX2-induced gastric cancers and are observed in lower frequency in the sub-set with SOX2-expression maintenance.

As a group, gastric cancers with mRNA CDX2 induction display differences both in the genomic alterations and in the immune presentation machinery compared with gastric cancers with no CDX2 mRNA induction [15,38]. The master regulator of HLA class II antigens CIITA and both variant and invariant HLA class II chains displayed a decreased mRNA expression in CDX2-induced gastric cancers compared with gastric cancers with no CDX2 induction. However, the two groups with differential expression of SOX2 within the CDX2-induced group did not differ significantly in their expression of HLA class II molecules, suggesting that CDX2 may be instrumental in suppressing this expression. SOX2 may play a role in inducing HLA class II expression as the group of CDX2-induced cancers with SOX2 maintenance had a slightly higher expression of these immune presenters. However, based on the small differences of the observed expressions, a major regulatory role for SOX2 is unlikely. HLA class II molecules are mostly expressed by professional immune presentation cells, such as dendritic cells and macrophages, but they can be expressed by some cancers, in which they have been associated with an improved prognosis [39]. HLA II molecules are able to present a wider range of antigens to helper T cells than the range of antigens HLA I present to cytotoxic T cells [40]. Low expression of HLA II molecules in CDX2-induced gastric cancers may contribute to a cold immune environment and may also be a contributing factor in the immune tolerance of *h. pylori*, which is one of the common inducers of gastric injury and intestinal metaplasia [41]. In addition, induction of HLA II could provide a therapeutic opportunity for increasing the effectiveness of immunotherapy. The current immunotherapy approaches with immune checkpoint inhibitors have limited efficacy in most gastric adenocarcinomas, which are MMR proficient [42]. Therefore, therapies converting immunologically cold tumors to immunologically hot tumors by inducing antigen presentation in combination with immune checkpoint inhibitors could be a way to unleash a more effective immunotherapy response [43].

The current study has a few notable limitations. The source series has examined several clinical characteristics in a substantial number of patients with gastric cancer but only a sub-set had CDX2 over-expression, reducing the eligible patients in this study. The study examined only one cohort of gastric cancers, and examination of additional cohorts would solidify the generalizability of the presented findings. In addition, some important clinical elements such as the presence of *h. pylori* infection and treatments received by the participating patients were not captured. Examination of series considering these important parameters in the pathogenesis and therapy of gastric cancer would be needed in future studies. The series examined mRNA expressions without a parallel evaluation of the protein expression of genes of interest. As such, it remains unclear whether the groups studied could be reliably defined using immunohistochemistry (IHC) in the clinical setting. In that case, a clinical translation of CDX2 and SOX2 as biomarkers of potentially targetable sub-sets of gastric cancers would be more straightforward, given that the infrastructure for IHC performance and evaluation exist widely in clinical pathology laboratories. Similar studies to the one presented here incorporating protein evaluation would be required to advance the translational value of the results. Finally, the study has not examined therapeutic interventions and was not designed to inform CDX2, SOX2, or any other alterations as predictive biomarkers of existing therapies received.

In conclusion, gastric cancers with CDX2 induction form two distinct groups according to SOX2 expression that could be used for guiding personalized targeted therapies. For example, the sub-set of gastric cancers with SOX2-suppressed expression and MSI instability could be targets for immunotherapy, possibly in combination with treatments that would induce antigen presentation by up-regulation of HLA II molecules. Given the association of CDX2 induction with the suppression of HLA II molecules, therapeutic approaches that prevent or reverse intestinal metaplasia and CDX2 induction may enhance the immunogenicity of gastric cancers. On the other hand, gastric cancers with CDX2 induction and SOX2-retained expression could benefit from future approaches targeting chromosome instability as a process. Kinase TTK1 (Threonine Tyrosine Kinase 1, also known as Mps1) is an integral part of the spindle assembly checkpoint which monitors the kinetochore attachment to microtubules during mitosis and prevents anaphase before all centromeres are attached [44]. Inhibitors of the mitotic spindle kinase TTK1 have been developed and function through accentuating chromosome desegregation. Cells with defects leading to increased chromosome instability may be particularly vulnerable to dysfunction of the spindle assembly checkpoint, as this would produce a further increase in aneuploidy which is incompatible with survival. Pre-clinical studies have shown that some gastric cell lines with chromosome instability are sensitive to TTK1 inhibitors, but sensitivity partially depends on the status of p53 [45]. Further studies would be needed to translate these mechanistic and pre-clinical insights to clinical therapeutic advances.

## Figures and Tables

**Table 1:** Clinical characteristics of the entire TCGA gastric cancer cohort with CDX2 induction and of the groups with SOX2-maintained expression (mRNA expression z score relative to normal samples above 0, *n* = 97) or suppressed SOX2 expression (mRNA expression z score relative to normal samples below 0, *n* = 123). NA: Not available, NOS: Not otherwise specified.

	All CDX2-Induced Patients (*n* = 240) (%)	CDX2-Induced and SOX2-Non-Suppressed Patients (*n* = 97) (%)	CDX2-Induced and SOX2-Suppressed Patients (*n* = 124) (%)	*p*
Age (mean)	66.5 ± 10.3	64.2 ± 10.3	67.7 ± 9.8	0.01
Early onset (≤50 years-old)				
yes	14 (5.9)	7 (7.4)	6 (4.9)	0.56
no	223 (94.1)	88 (92.6)	117 (95.1)	
NA	3	2	1	
Sex				
Male	157 (65.4)	62 (63.9)	83 (66.9)	0.67
Female	83 (34.6)	35 (36.1)	41 (33.1)	
Histology				
Intestinal/NOS adenocarcinoma	132 (55)	51 (52.6)	66 (53.2)	0.68
Diffuse	27 (11.2)	12 (12.4)	11 (8.9)	
Other	81 (33.8)	34 (35)	47 (37.9)	
Grade				
1–2	110 (46.6)	45 (47.9)	60 (48.8)	1
3	126 (53.4)	49 (52.1)	63 (51.2)	
NA	4	3	1	
T stage				
T1	13 (5.5)	2 (2.1)	11 (8.9)	0.16
T2	57 (24.2)	21 (21.6)	28 (22.8)	
T3	102 (43.2)	48 (49.5)	51 (41.5)	
T4	64 (27.1)	26 (26.8)	33 (26.8)	
NA	4	0	1	
N stage				
N0	74 (31.8)	20 (21.1)	49 (40.2)	0.02
N1	66 (28.3)	31 (32.6)	31 (25.4)	
N2	48 (20.6)	21 (22.1)	23 (18.8)	
N3	45 (19.3)	23 (24.2)	19 (15.6)	
NA	7	2	2	
M stage				
M0	217 (94.8)	90 (95.7)	111 (95.7)	1
M1	12 (5.2)	4 (4.3)	5 (4.3)	
NA	11	3	8	
Stage				
I	35 (15.1)	8 (8.3)	23 (19)	0.04
II	77 (33.2)	30 (31.2)	45 (37.2)	
III	102 (44)	52 (54.2)	46 (38)	
IV	18 (7.7)	6 (6.3)	7 (5.8)	
NA	8	1	3	

**Table 2:** Genomic characteristics of the entire TCGA gastric cancer cohort with CDX2 induction and of the groups with SOX2-maintained expression (mRNA expression z score relative to normal samples above 0, *n* = 97) or suppressed SOX2 expression (mRNA expression z score relative to normal samples below 0, *n* = 123). CIN: Chromosomal Instability, MSI: Microsatellite Instability, GS: Genomically Stable, EBV: Epstein–Barr Virus, POLE: Polymerase epsilon, TMB: Tumor Mutation Burden, AS: Aneuploidy Score, NA: Not available.

	All CDX2-Induced Patients (*n* = 240) (%)	CDX2-Induced and SOX2-Maintained Patients (*n* = 97) (%)	CDX2-Induced and SOX2-Suppressed Patients (*n* = 124) (%)	*p*
Genomic category				
CIN	138 (63.3)	67 (74.5)	60 (54.6)	0.003
GS	25 (11.5)	10 (11.1)	13 (11.8)	
MSI	47 (21.5)	9 (10)	34 (30.9)	
EBV	3 (1.4)	2 (2.2)	0	
POLE	5 (2.3)	2 (2.2)	3 (2.7)	
NA	22	7	14	
TMB				
High (>10 mutations/Mb)	59 (24.8)	12 (12.4)	43 (35)	0.0001
Low (≤10 mutations/Mb)	179 (75.2)	85 (87.6)	80 (65)	
NA	2		1	
AS				
<4	82 (35.1)	12 (12.9)	43 (35.3)	<0.00001
4–24	136 (58.1)	67 (72)	77 (63.1)	
>24	16 (6.8)	14 (15.1)	2 (1.6)	
NA	6	4	2	
FGA				
<0.08	63 (26.5)	18 (18.7)	40 (32.5)	0.03
>0.08	175 (73.5)	78 (81.3)	83 (67.5)	
NA	2	1	1	

**Table 3:** Mutations in epigenetic modifiers in samples with CDX2 induction and in the groups with SOX2-maintained expression (mRNA expression z score relative to normal samples above 0, *n* = 97) or suppressed SOX2 expression (mRNA expression z score relative to normal samples below 0, *n* = 123). The sum of the mutations in each gene does not equal the total number of mutations in the whole cohort of patients because some samples did not have the required mRNA expression data to be categorized into the two groups.

Gene	CDX2-Induced Patients (*n* = 238) (%)	CDX2-Induced and SOX2-Maintained Patients (*n* = 97) (%)	CDX2-Induced and SOX2-Suppressed Patients (*n* = 123) (%)	*p*	q
ARID2	18 (7.6)	4 (4.1)	13 (10.6)	0.12	1
ARID1A	59 (24.8)	16 (16.5)	37 (30.1)	0.02	0.4
ARID1B	14 (5.9)	5 (5.2)	7 (5.7)	1	1
ARID5B	11 (4.6)	1 (1)	8 (6.5)	0.08	1
KMT2C	35 (14.7)	5 (5.2)	25 (20.3)	0.001	0.02
KMT2D	38 (16)	7 (7.2)	28 (22.8)	0.001	0.02
KMT2A	29 (12.2)	7 (7.2)	19 (15.4)	0.09	1
KMT2B	30 (12.6)	5 (5.2)	21 (17.1)	0.006	0.12
DNMT3A	7 (2.9)	2 (2.1)	4 (3.3)	0.69	1
DNMT1	9 (3.8)	1 (1)	8 (6.5)	0.08	1
DNMT3B	6 (2.5)	2 (2.1)	4 (3.3)	0.69	1
KDM5C	7 (2.9)	1 (1)	5 (4.1)	0.23	1
KDM6A	10 (4.2)	3 (3.1)	5 (4.1)	1	1
KDM5A	11 (4.6)	2 (2.1)	8 (6.5)	0.19	1
EP300	13 (5.5)	4 (4.1)	8 (6.5)	0.55	1
CREBBP	27 (11.3)	9 (9.3)	17 (13.8)	0.4	1
SETD2	12 (5)	2 (2.1)	10 (8.1)	0.07	1
SMARCA2	14 (5.9)	2 (2.1)	10 (8.1)	0.07	1
SMARCA4	16 (6.7)	5 (5.2)	10 (8.1)	0.43	1
SMARCB1	8 (3.4)	0	7 (5.7)	0.01	0.2

**Table 4:** Mutations in receptor tyrosine kinases in samples with CDX2 induction and in the groups with SOX2-maintained expression (mRNA expression z score relative to normal samples above 0, *n* = 97) or suppressed SOX2 expression (mRNA expression z score relative to normal samples below 0, *n* = 123). The sum of the mutations in each gene does not equal the total number of mutations in the whole cohort of patients because some samples did not have the required mRNA expression data to be categorized into the two groups.

Gene	CDX2-Induced Patients (*n* = 238) (%)	CDX2-Induced and SOX2-Non-Suppressed Patients (*n* = 97) (%)	CDX2-Induced and SOX2-Suppressed Patients (*n* = 123) (%)	*p*
EGFR	14 (5.9)	5 (5.2)	9 (7.3)	0.58
ERBB2	16 (6.7)	6 (6.2)	8 (6.5)	1
ERBB3	26 (10.9)	8 (8.2)	16 (13)	0.28
ERBB4	31 (13)	7 (7.2)	21 (17.1)	0.04
FGFR1	8 (3.4)	0	7 (5.7)	0.01
FGFR2	8 (3.4)	2 (2.1)	4 (3.3)	0.69
FGFR3	7 (2.9)	1 (1)	5 (4.1)	0.23
FGFR4	9 (3.8)	4 (4.1)	5 (4.1)	1
PDGFRA	9 (3.8)	4 (4.1)	5 (4.1)	1
PDGFRB	12 (5)	2 (2.1)	8 (6.5)	0.19
NTRK1	6 (2.5)	1 (1)	4 (3.3)	0.38
NTRK2	13 (5.5)	4 (4.1)	8 (6.5)	0.55
NTRK3	11 (4.6)	3 (3.1)	8 (6.5)	0.35
ALK	12 (5)	4 (4.1)	6 (4.9)	1
ROS1	16 (6.7)	4 (4.1)	11 (8.9)	0.18
VEGFR1	0	0	0	
VEGFR2	0	0	0	
VEGFR3	0	0	0	
VEGFR4	0	0	0	
MET	5 (2.1)	0	5 (4.1)	0.06
RET	10 (4.2)	2 (2.1)	7 (5.7)	0.3
INSR	9 (3.8)	2 (2.1)	6 (4.9)	0.47
IGF1R	13 (5.5)	1 (1)	9 (7.3)	0.04
EPHA1	10 (4.2)	3 (3.1)	6 (4.9)	0.73
EPHB1	16 (6.7)	4 (4.1)	10 (8.1)	0.27

**Table 5:** Mutations in DNA damage response (DDR) genes in samples with CDX2 induction and in the groups with SOX2-maintained expression (mRNA expression z score relative to normal samples above 0, *n* = 97) or suppressed SOX2 expression (mRNA expression z score relative to normal samples below 0, *n* = 123). The sum of the mutations in each gene does not equal the total number of mutations in the whole cohort of patients because some samples did not have the required mRNA expression data to be categorized into the two groups.

Gene	CDX2-Induced Patients (*n* = 238) (%)	CDX2-Induced and SOX2-Non-Suppressed Patients (*n* = 97) (%)	CDX2-Induced and SOX2-Suppressed Patients (*n* = 123) (%)	*p*
BRCA1	8 (3.4)	0	7 (5.7)	0.01
BRCA2	18 (7.6)	4 (4.1)	12 (9.8)	0.12
PALB2	7 (2.9)	0	7 (5.7)	0.01
RAD51	1 (0.4)	0	1 (0.8)	1
RAD51B	0	0	0	1
RAD51C	1 (0.4)	0	1 (0.8)	1
RAD51D	1 (0.4)	1 (1)	0	0.44
RAD50	5 (2.1)	2 (2.1)	2 (1.6)	1
XRCC2	3 (1.3)	1 (1)	1 (0.8)	1
ATM	24 (10.1)	6 (6.2)	17 (13.8)	0.07
ATR	12 (5)	3 (3.1)	8 (6.5)	0.35
BRIP1	4 (1.7)	1 (1)	3 (2.4)	0.63
NBN	9 (3.8)	2 (2.1)	6 (4.9)	0.47
MRE11	5 (2.1)	1 (1)	4 (3.3)	0.38
CHEK1	5 (2.1)	1 (1)	4 (3.3)	0.38
CHEK2	6 (2.5)	1 (1)	4 (3.3)	0.38
BARD1	12 (5)	3 (3.1)	6 (4.9)	0.73
BAP1	8 (3.4)	2 (2.1)	6 (4.9)	0.47
POLQ	19 (8)	6 (6.2)	12 (9.8)	0.45
CDK12	10 (4.2)	3 (3.1)	7 (5.7)	0.51

**Table 6:** Mutations in MMR genes and genes encoding for polymerases POLE and POLD1 in the groups with SOX2-maintained expression (mRNA expression z score relative to normal samples above 0, *n* = 97) or suppressed SOX2 expression (mRNA expression z score relative to normal samples below 0, *n* = 123).

Gene	CDX2-Induced Patients (*n* = 238) (%)	CDX2-Induced and SOX2-Non-Suppressed Patients (*n* = 97) (%)	CDX2-Induced and SOX2-Suppressed Patients (*n* = 123) (%)	*p*
MSH2	7 (2.9)	2 (2.1)	4 (3.3)	0.69
MSH6	8 (3.4)	3 (3.1)	4 (3.3)	1
MLH1	5 (2.1)	4 (4.1)	1 (0.8)	0.17
PMS2	6 (2.5)	1 (1)	4 (3.3)	0.38
POLE	19 (8)	4 (4.1)	13 (10.6)	0.12
POLD1	9 (3.8)	2 (2.1)	7 (5.7)	0.3

## Data Availability

No data beyond the data contained in the article are available.

## Supplementary Materials

The following supporting information can be downloaded at: https://www.mdpi.com/article/10.3390/genes16030279/s1, Figure S1: Distribution of mRNA expressions of CDX2 and SOX2 in gastric cancer. Data are from the Cancer Genome Atlas.
