# Supplementary figures and images for: The Status of SOX2 Expression in Gastric Cancers with Induction of CDX2 Defines Groups with Different Genomic Landscapes
Source: Genes (Basel). 2025 Feb 26;16(3):279. doi: 10.3390/genes16030279 (PMC11942492; doi:10.3390/genes16030279)

## Supplemental Figure S1

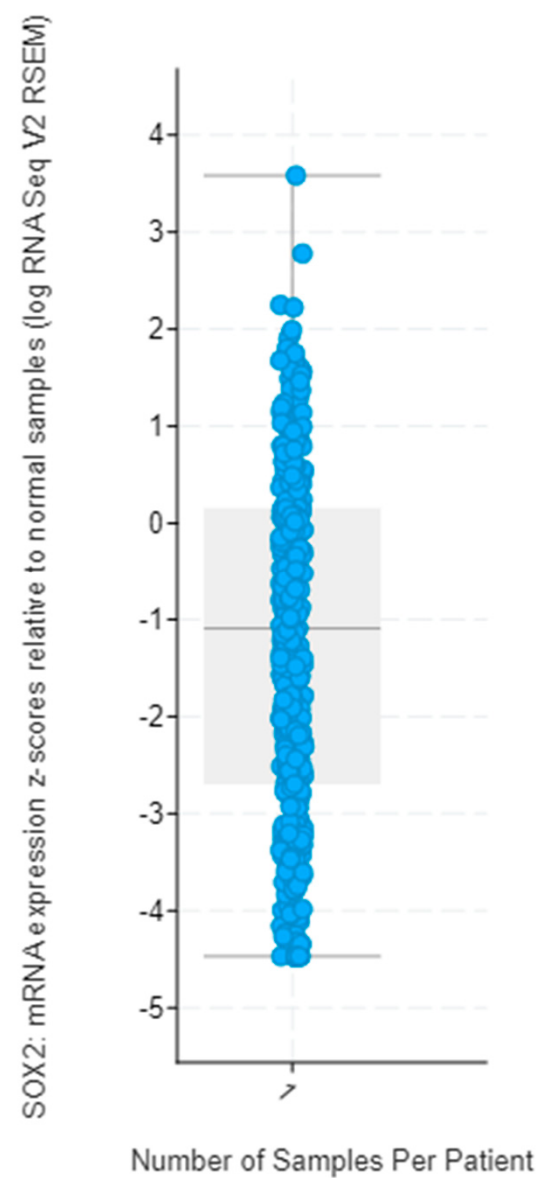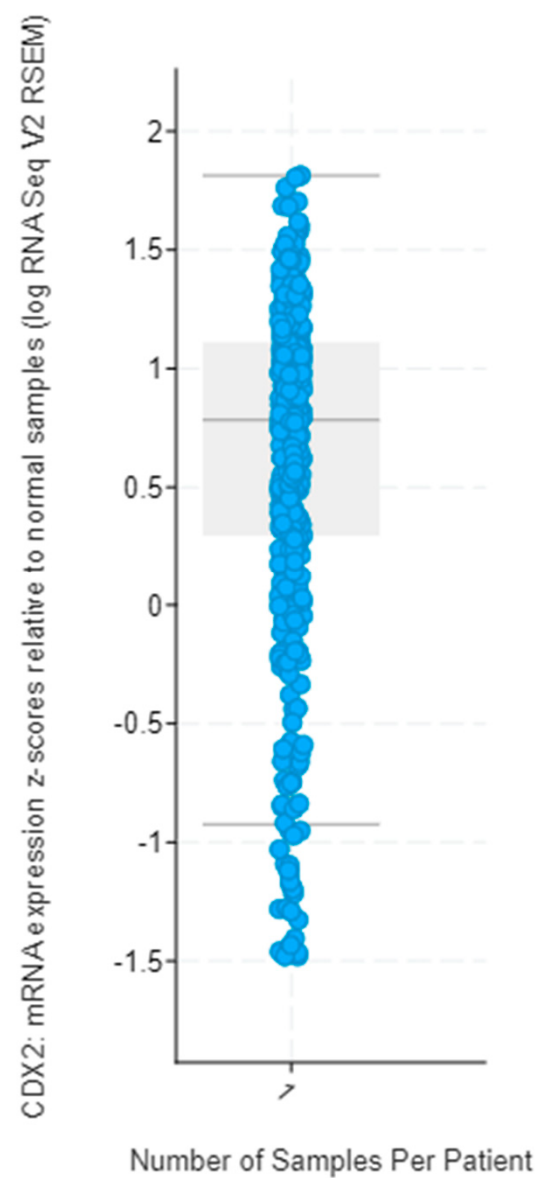

Supplement: Supplementary file 1 [file genes-16-00279-s001.zip › genes-3495421-supplementary.pdf]
